# Retracted: Propofol Causes Consciousness Loss by Affecting GABA-A Receptor in the Nucleus Basalis of Rats

**DOI:** 10.1155/2022/9840256

**Published:** 2022-02-21

**Authors:** Behavioural Neurology

*Behavioural Neurology* and the authors have retracted the article titled “Propofol Causes Consciousness Loss by Affecting GABA-A Receptor in the Nucleus Basalis of Rats” [1], due to concerns with the methodology which invalidates the conclusions. Following publication of the article, the authors identified concerns that the use of sodium pentobarbital as an anaesthetic may interfere with the function of propofol due to its interaction with the GABA-A receptor [2, 3]. The conclusions drawn in the article are therefore considered unreliable.

The authors apologize for this error and agree to the retraction of the article, which is also with the agreement of the editorial board.

## References

[1] Xing Y., Li K., Jiao Y., Li Z. (2020). Propofol Causes Consciousness Loss by Affecting GABA-A Receptor in the Nucleus Basalis of Rats. *Behavioural Neurology*.

[2] Sierra-Valdez F. J., Ruiz-Suárez J. C., Delint-Ramirez I. (2016). Pentobarbital modifies the lipid raft-protein interaction: A first clue about the anesthesia mechanism on NMDA and GABA_A_ receptors. *Biomembranes*.

[3] MacDonald R. L., Rogers C. J., Twyman R. E. (1989). Barbiturate regulation of kinetic properties of the GABAA receptor channel of mouse spinal neurones in culture. *The Journal of Physiology*.

